# Implementation of an antenatal magnesium sulfate protocol for fetal neuroprotection in preterm infants

**DOI:** 10.1038/srep14732

**Published:** 2015-09-29

**Authors:** Pierre-Emmanuel Bouet, Stéphanie Brun, Hugo Madar, Anne-Laure Baisson, Véronique Courtay, Géraldine Gascoin-Lachambre, Sigismond Lasocki, Loïc Sentilhes

**Affiliations:** 1 Department of Obstetrics and Gynecology, Montreal University Hospital—Montreal—Canada; 2 Department of Obstetrics and Gynecology, Bordeaux University Hospital—Bordeaux—France; 3 Department of Obstetrics and Gynecology, Angers University Hospital—Angers—France; 4 Department of Neonatal Medicine, Angers University Hospital—Angers—France; 5 Department of Anesthesiology, Angers University Hospital—Angers—France

## Abstract

The aim of our study was to assess the feasibility of implementing a protocol for the use of magnesium sulfate to prevent cerebral palsy. This retrospective single-center study included all women with fetuses of gestational age <33 weeks of gestation whose birth was planned or expected within 24 hours from September 2011 to December 2012. They were to receive magnesium sulfate, administered intravenously as a 4-g bolus followed by a constant infusion of 1 g per hour. If delivery had not occurred after 12 hours and was no longer considered imminent, the infusion was to be discontinued. The study included 119 women, 81 (68.1%) of whom received magnesium sulfate. Among the latter, 71 (87.5%) gave birth within 24 hours. The reasons treatment was not given were: omission by medical team (19/38, 50%), urgent delivery (18/38, 47.4%), and contraindication to treatment (1/38, 2.6%). The mean gestational age at protocol implementation was 29.6 +/− 2.1 weeks. Maternal monitoring, especially at the onset of infusion, appeared suboptimal. No major maternal side effects were observed. Our study shows that implementing a protocol for prevention of cerebral palsy by magnesium sulfate is feasible in a tertiary obstetric center.

The incidence of preterm birth in developed countries is estimated at 7.5% of live births^1^. The survival of infants born preterm is associated with a substantial risk of neurodevelopmental impairment. Cerebral palsy (CP) is the most frequent cause of childhood motor disability, affecting approximately 2 per 1,000 live births in high-income countries^2^. The risk of CP is higher at earlier gestational ages, and 30%–50% of the children with CP were born preterm^3^.

Magnesium sulfate is involved in many intracellular processes, acting to induce cerebral vasodilatation, reduce inflammatory cytokines and oxygen free radicals, and inhibit calcium influx into cells^4^. Animal studies have shown that it has a neuroprotective effect^5^ and that it may also interact with antenatal steroids to preserve the integrity of the blood-brain barrier in neuroinflammation^6^.

Five randomized controlled trials (RCTs), published between 2002 and 2008^7^,^8^,^9^,^10^,^11^ and including 6145 infants, have demonstrated that intravenous magnesium sulfate given to mothers before early preterm birth significantly decreases the risk of CP without increasing perinatal or infant mortality. A meta-analysis^12^ examining these 5 RCTs reported that, among the infants surviving to 18–24 months of age, the number needed to treat to prevent one case of CP is 46 when magnesium sulfate is administered in utero before 30 weeks and 56 when administered before 32 to 34 weeks.

Its administration is therefore recommended by United States and Canadian guidelines for women with imminent preterm birth^13^,^14^.

The primary objective of the study was to assess the feasibility of implementing a protocol for the use of magnesium sulfate among gravidas at imminent risk of delivery before 33 weeks of gestation, to prevent CP in their babies. As a secondary objective, we sought to examine maternal safety and the reasons for non-compliance with the protocol in daily practice.

## Materials and Methods

This retrospective single-center study took place from September 2011 (when the magnesium sulfate protocol was implemented in our department) to December 2012 at the Obstetrics and Gynecology department of the Angers University Hospital, a tertiary care referral center with approximately 4,100 births/annum.

All women with singleton, twin, or triplet pregnancies were eligible for the protocol if they were admitted with a viable fetus at less than 33 weeks of gestation (Appendix). Gestational age was the best estimate of completed weeks of gestation based on early ultrasound and menstrual history, as recommended by the French College of Gynecologists and Obstetricians guidelines^15^. For women with imminent preterm birth between 24 and 33 weeks of gestation, antenatal magnesium sulfate is to be administered as a 4-g IV loading dose, over 30 minutes, followed by a 1 g/hour maintenance infusion until birth (for a maximum of 12 hours)^8^. Imminent preterm birth is defined as high likelihood of birth due to either active labor with cervical dilatation ≥4 cm, with or without preterm premature rupture of membranes (PPROM), or planned preterm birth for fetal or maternal indications or other situations such as significant vaginal bleeding.

In our protocol (Appendix), the relative contraindications for magnesium sulfate are: electrolyte disorders, renal failure, defined as rapidly progressive loss of renal function characterized by oliguria (quantified as less than 400 mL per day), maternal cardiac arrhythmia during this pregnancy, myasthenia, ingestion of calcium channel blockers during the previous 2 hours, and “urgent delivery” (i.e., sulfate administration is allowed in this type of indication as long as it does not delay cesarean delivery).

We considered “urgent delivery” any maternal or fetal emergency that required delivery in the shortest time such as abnormal fetal heart rate tracing, severe antepartum hemorrhage or abruptio placenta.

Our study included all women who were admitted for preterm labor and induced preterm birth (fetal or maternal indications) for delivery between 24 and 33 weeks of gestation, regardless of whether magnesium sulfate was contraindicated. Exclusion criteria were major fetal abnormalities or intrauterine fetal death between 24 and 33 weeks and incomplete medical records.

Women with severe preeclampsia could receive magnesium sulfate for cerebral palsy and seizure prophylaxis as recommended by the French Society of Anesthesiologist and Intensive Care^16^.

The protocol was then drafted jointly by obstetricians, anesthesiologists, and pediatricians. We held two further multidisciplinary meetings before starting to implement it, to explain its terms and underline its interest once again to the entire all health care team. The protocol was then sent by email to the entire medical team (attending and regular obstetricians, anesthesiologists, pediatricians and midwives).

We decided to begin the protocol at a time of year when few medical staff members are on vacation (September).

During the first month of implementation, the supervising midwives regularly reminded all staff working on the maternity ward that the detailed protocol was available in the Standard Operating Procedure folder (digital and hard copy), to refer to whenever needed.

The primary outcome for this study was the proportion of women who delivered before 33 weeks of gestation and received magnesium sulfate before delivery (at least the 4-g IV loading dose). Based on the three studies^17^,^18^,^19^ assessing the feasibility of implementing a magnesium sulfate protocol for neuroprotection, and organizational commitment in general, we considered that the implementation procedure would be successful if 60% of eligible patients received the treatment during the study period.

The main secondary outcomes were maternal safety, assessed by the occurrence of potentially magnesium-attributable maternal or perinatal complications, and reasons for non-compliance.

Women and infants were cared for according to standard clinical guidelines. The protocol calls for recording maternal pulse rate, blood pressure, respiratory rate, tendon reflexes, and any adverse effects throughout the infusion. Fetal heart rate was monitored throughout labor.

According to our protocol, treatment is to be stopped in the presence of the following symptoms: respiratory rate <10/min, hypotension, areflexia, disorders of consciousness, or oliguria/anuria.

Mothers and their children were followed up until hospital discharge.

Neonatal medical records were reviewed to identify the following outcomes: neonatal death; 5-minute Apgar score <7; intraventricular hemorrhage (IVH) grades 3 and 4, according to cranial ultrasound findings and Papile’s criteria^20^; periventricular leukomalacia, defined as hyperechoic lesions persisting to day 7 of life^21^, and necrotizing enterocolitis grades II/III according to Bell’s classification^22^.

Informed consent was obtained from all participants. All experimental protocol was approved by the institutional review committee of the Angers University Hospital. This study was conducted in accordance with the approved guidelines.

Data analysis used χ2 or Fisher’s exact test, as appropriate, for categorical variables and the ANOVA test for continuous variables, with the SPSS statistical software program package (SPSS version 15.0 for Windows, SPSS Inc., Chicago, IL). Statistical significance was defined as a *P* value < 0.05.

## Results

**Figure 1:**
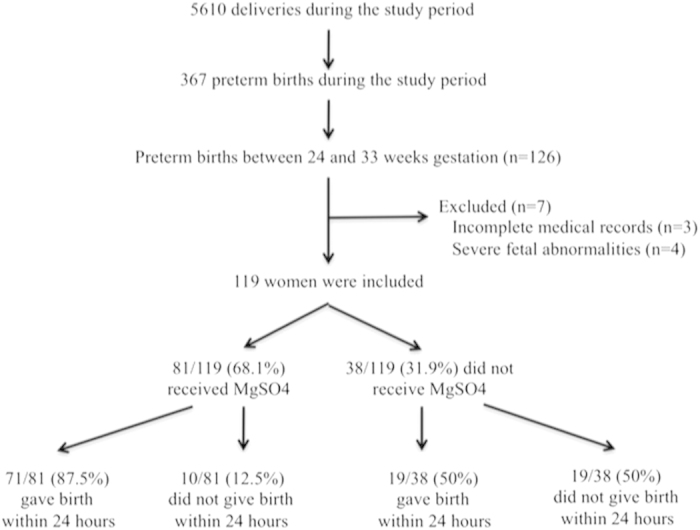
Flow diagram showing the study organization.

Of the 5610 women who delivered during the study period, 126 (2.2%) were eligible for the protocol. They accounted for 34.3% (126/367) of all preterm births; the other 241 preterm births were ineligible because they occurred between 33 and 37 weeks of gestation. After 7 women, 3 with incomplete medical records and 4 with severe fetal abnormalities, were excluded, the study finally included 119 women and 138 premature newborns. Among these women, 81 (68.1%) received magnesium sulfate (Fig. 1), and 71 of these 81 (87.5%) then gave birth within 24 hours.

In 89% (72/81) of cases, the magnesium sulfate protocol was ordered by the on-call obstetrician, and in another 6 cases (7.4%), by consensus during the daily morning staff meeting.

In the 3 months immediately after the protocol was implemented, 19 of 25 eligible women (76%, 95% confidence interval (CI) 54.9–90.6) received magnesium sulfate before delivery compared with 87.5% (95% CI 47.3–99.7) in the final 3 months of the study period (p = 0.49).

**Figure 2:**
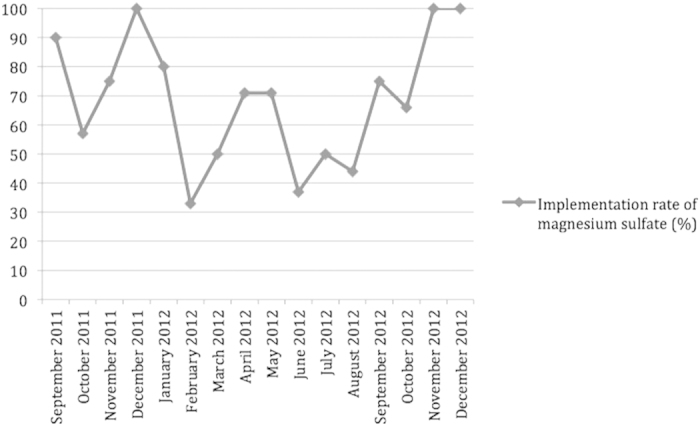
Implementation rate of magnesium sulfate.

In the 3 months of the summer period (June, July and August 2012), 13 of 29 eligible women (45%, 95% CI 26.7–62.9) received magnesium sulfate compared with 76% in the 3 months following the implementation of the protocol (p = 0.01) and 87.5% in the final 3 months of the study period (p = 0.04) (Fig. 2).

Mean gestational age (GA) at magnesium sulfate administration was 29.6 weeks +/− 2.1 days (24 weeks + 3 days to 32 weeks + 6 days). The mean duration of therapy was 285 +/− 40 minutes, and the mean duration of bolus 34 min (+/− 11 min). Sixty women (74%) received maintenance infusions with a mean duration of 333 +/− 79 min. The total mean dose administered was 8 +/− 6.7 g. The median total predelivery dose was 5.5 g (range: 0.9 g to 39.5 g). Four women (3.3%) received magnesium sulfate for more than 12 hours, which violated the protocol.

The women’s characteristics are described in Table 1. The main reasons for preterm birth were preterm labor, PPROM, prepartum hemorrhage, and preeclampsia (Table 1). There were 5 (5/81; 6%) women with severe preeclampsia who received magnesium sulfate for cerebral palsy and seizure prophylaxis.

The reasons that indicated treatment did not occur were omission by medical team (19/38, 50%), “urgent delivery” (18/38, 47.4%), and contraindication to treatment (1/38, 2.6%). “Urgent delivery” included: category III^19^ fetal heart rate tracings (15/38, 39.5%), severe antepartum hemorrhage due to placenta previa (2/38, 5.3%) and abruptio placenta (1/38, 2.6%) (Table 1).

If we excluded the women with relative contraindications (“urgent delivery” (n = 18) and maternal arrhythmia (n = 1)), 81% (81/100) of the women included received magnesium sulfate.

Adverse effects were recorded for 20 (24.7%) of the 81 women treated. They were mainly hypotension (45%, 9/20), flushing or sweating (20%, 4/20), nausea or vomiting (10%, 2/20), headache (5%, 1/20), palpitation (5%, 1/20) and hyporeflexia (5%, 1/20) (Table 1). Magnesium sulfate administration was stopped in 3 women (3.7%): 1 with hyporeflexia, 1 with respiratory depression, and 1 with hypotension. Among women who did not receive magnesium sulfate, only three (7.9%) had symptoms: nausea (n = 1), flushing (n = 1), and hypotension (n = 1) (Table 1).

Rates of emergency delivery, abnormal fetal heart rate, general anesthesia, neonatal cord pH < 7.10 at birth, Apgar score at 5 minutes <7, neonatal external cardiac massage, and use of epinephrine were significantly higher in the group of women who did not receive magnesium sulfate before birth, and the rate of antenatal corticoid administration was significantly lower in this group (Tables 1 and 2).

The rates of neonatal mortality before hospital discharge tended to be lower in infants whose mothers received magnesium sulfate, but the difference was not significant (Table 2). No neonatal adverse effects were recorded.

When bolus administration began, blood pressure was monitored in 63% (51/81) of the women, heart rate in 72.8% (59/81), respiratory rate in 25.9% (21/81), oxygen saturation in 8.6% (7/81), and patellar reflexes in 23.5% (19/81) (Table 3). Four hours after administration began, among the women who had not delivered and were still receiving the infusion (n = 26), blood pressure was being monitored in 73% (19/26), heart rate in 65.4% (17/26), respiratory rate in 42.4% (11/26), oxygen saturation in 26.9% (7/26), and patellar reflexes in 57.7% (15/26) (Table 3). The rate of monitoring related to the measurement of respiratory rate, oxygen saturation, and patellar reflexes thus increased significantly during the course of the infusion (Table 3).

When magnesium sulfate administration began, all characteristics to be monitored were actually mentioned as checked in the records of only 3.7% of women (3/81) compared with 19.2% (5/26, including the initial 3) four hours later (p < 0.05). Fifty-five women gave birth within 4 hours.

## Discussion

This study demonstrates the feasibility of implementing a protocol to use magnesium sulfate among gravidas at imminent risk of delivery before 33 weeks of gestation to prevent cerebral palsy. Appropriate selection of women at high risk of imminent preterm birth is feasible. Overall, 68.1% of the eligible women and 81% of those without any contraindications received magnesium sulfate before preterm delivery, with a high rate from the outset of the protocol’s implementation (76% during the first three months).

In the months leading up to the implementation of our new protocol, we first organized a multidisciplinary meeting (midwives, obstetricians, anesthesiologists, and pediatricians) to discuss the recently issued guidelines^13^,^14^ and the level of evidence for the use of magnesium sulfate for neuroprotection in preterm infants and its possible side effects.

The maternal-fetal medicine department and the labor room quickly adopted the use of magnesium sulfate to prevent cerebral palsy.

The significant differences between the groups with and without magnesium sulfate treatment for maternal and neonatal characteristics (i.e., abnormal fetal heart rate, general anesthesia, neonatal cord pH < 7.10 at birth, Apgar score at 5 minutes < 7, neonatal external cardiac massage, use of epinephrine, and antenatal corticoid administration) strongly suggest that “urgent delivery” for fetal indication was much more frequent in the untreated group.

Overall, 15 women did not receive magnesium sulfate because fetal distress required immediate emergency delivery. However, only 8 of the 15 were admitted for preterm labor with a category III fetal heart tracing noted upon emergency admission and did not receive antenatal corticosteroids. The remaining seven had been hospitalized for preterm labor for several days and could have received antenatal corticosteroids before the emergency cesarean delivery for fetal distress.

To our knowledge, only three studies^17^,^18^,^19^ have assessed the feasibility and maternal safety of implementing the use of magnesium sulfate for neuroprotection. Ow *et al.*^17^ reported a 40% rate in the first 12 months after implementation of their guideline. The only information available in that study related to potentially magnesium-attributable maternal complications was that infusion was discontinued as result of side effects in 2% of women, mostly for hypotension. Gibbins *et al.*^18^ and, more recently, Tan *et al.*^19^, implemented a similar departmental guideline and respectively reported a 51% and 82% implementation rate during the first year. Like us, they reported no episodes of severe maternal adverse effects.

We observed a significant decrease of the implementation rate of magnesium sulfate during the summer (45%). This is mainly due to the significant reduction in staff observed during that period, since many doctors and residents take their vacations in the July-August period. It is important to note that this is not due to the “July phenomenon” frequently referred to in the medical literature^23^—where the arrival of new interns and residents at teaching hospitals each July is thought to cause an annual transient increase in poor patient outcomes and ineffective care—since in France, new residents and fellows start working in November. However, our department had many newly graduated midwives working on the maternity ward during that period, and this might have influenced the implementation rate.

Clinical monitoring of women who receive magnesium sulfate is essential, especially because the infusion may need to be interrupted because of an adverse event. In our study, administration was interrupted in 3 patients (3.7%) (1 for hyporeflexia, 1 for respiratory depression and 1 for hypotension). These results are consistent with the literature^10^,^11^,^24^.

Nevertheless, our clinical monitoring of women who received magnesium sulfate was clearly suboptimal because only 3.7% (3/81) of patients were adequately monitored during the bolus. It does appear that the medical team was aware of the necessity to monitor because this rate was significantly higher 4 hours after the beginning of the infusion (19.2%; 5/26) (p < 0.05). It is also possible, especially in cases when the delivery may be considered likely to become urgent, that magnesium sulfate administration is the highest priority, and monitoring is set up afterwards.

The fear of suboptimal maternal monitoring in daily practice is one of the main arguments made by those opposed to this neuroprotective procedure. Although we observed no serious adverse maternal effects, our study did show that close maternal monitoring is difficult to achieve in daily practice. Nevertheless, in our opinion, our result should be interpreted as an alert to the necessity of special attention to this monitoring rather than an argument against using magnesium sulfate.

Similarly, we observed other types of non-compliance with our protocol. In fact, we found that in 10 cases (12.3%), magnesium sulfate administration continued beyond the 12 hour maximum recommended by our protocol. The suboptimal clinical surveillance and the risk of non-compliance with the protocol in daily practice are arguments in favor of regular monitoring of the practical application of the protocol. Repeated audits should be conducted to alert and encourage medical teams to achieve these safety goals.

One way to improve safety outcomes may be to use the patient safety checklist recommended by the American College of Obstetricians and Gynecologists^25^, which enables the health care provider to review the inclusion and exclusion criteria and information for the woman (maternal side effects, neonatal benefits) and suggests different treatment regimens.

Our study has some limitations. The first is its retrospective design. Accordingly, all the flaws of retrospective analyses apply. In particular, there may be a problem of identifying patients or of data reliability. For example, monitoring might have been performed but not noted in the medical records. Finally, we underline that our study lacks the statistical power to draw a solid conclusion about adverse outcomes and neonatal benefits with magnesium sulfate use, but also note that such results were not the purpose of our study.

In conclusion, our study shows that implementing a protocol for prevention of cerebral palsy by magnesium sulfate is feasible in a tertiary obstetric center. However, maternal monitoring in our daily practice was suboptimal. This should alert physicians to the necessity to implement tools, such as audits or use of a patient safety checklist, to improve monitoring quality.

## Additional Information

**How to cite this article**: Bouet, P.-E. *et al.* Implementation of an antenatal magnesium sulfate protocol for fetal neuroprotection in preterm infants. *Sci. Rep.* **5**, 14732; doi: 10.1038/srep14732 (2015).

## Supplementary Material

Supplementary Information

## Figures and Tables

**Table 1:** Baseline characteristics of patients admitted with threatened or indicated delivery before 33 weeks of gestation.

	Magnesium sulfate (n = 81)	No magnesium sulfate (n = 38)	*P* value
Gestational age (weeks)^1^	29.6 +/− 2.1	30.1 +/− 1.9	0.5
Maternal age (years)^1^	29.3 +/− 6.2	30.3 +/− 4.2	0.6
Nulliparous, n (%)	40 (49.4)	14.6 (36.8)	0.2
Received antenatal corticotherapy, n (%)	77 (95.1)	30 (78.9)	<0.01
Abnormal fetal heart rate^2^, n (%)	28 (29.8)	21 (47.7)	<0.05
Principal reasons for preterm birth^3^			
• Preterm labor, n (%)	33 (41.3)	16 (44.4)	0.75
• Premature rupture of membranes, n (%)	22 (27.5)	15 (41.7)	0.13
• Antepartum hemorrhage, n (%)	19 (23.8)	9 (25)	0.9
• Preeclampsia, n (%)	29 (35.8)	4 (10.5)	<0.01
• Fetal growth restriction, n (%)	14 (17.5)	7 (19.4)	0.8
• Abruptio placenta, n (%)	4 (5)	4 (11.1)	0.23
• HELLP syndrom, n (%)	12 (15)	1 (2.8)	0.05
• Placenta prævia, n (%)	7 (8.6)	3 (8.3)	0.94
Use of tocolytic therapy, n (%)	41 (50.6)	16 (42.1)	0.4
Cesarean delivery, n (%)	52 (64.2)	24 (63.2)	0.9
General anesthesia, n (%)	11 (13.6)	14 (36.8)	<0.01
Adverse event^4^			
• Major side effects^5^, n (%)	0	0	−
• Flushing, sweating, n (%)	4 (4.9)	1 (2.6)	0.57
• Palpitation, n (%)	1 (1.2)	0	0.9
• Nausea or vomiting, n (%)	2 (2.5)	1 (2.6)	0.96
• Headache, n (%)	1 (1.2)	0	0.9
• Hypotension^6^, n (%)	9 (11.1)	1 (2.6)	0.15
• Respiratory depression^7^, n (%)	1 (1.2)	0	0.9
• Hyporeflexia, n (%)	1 (1.2)	0	0.9
• Reduction of respiratory frequency, n (%)	1 (1.2)	0	0.9
Relative contraindication to magnesium sulfate			
• Fetal emergency^8^, n (%)	13 (16.3)	15 (41.7)	<0.01
• Maternal emergency, n (%)	5 (6.3)	3 (8.3)	0.7
• Renal failure, n (%)	1 (1.2)	0	0.2
• Maternal arrythmia, n (%)	0	1 (2.6)	0.2

^1^Values are given as mean +/− standard deviation.

^2^Categories III of fetal heart rate tracings according to American College of Obstetricians & Gynecologists, Society for Maternal-Fetal Medicine and National Institute of Children’s Health and Development guidelines^26^.

^3^Some patients had more than one reason for preterm birth.

^4^Some patients had more than one adverse event.

^5^Respiratory rate of <10/min, diastolic blood pressure decrease of >30 mmHg, areflexia, coma, lung and heart failure^27^.

^6^Diastolic blood pressure decrease of more than 15 mmHg^26^.

^7^Decrease of >4/min from baseline^26^.

^8^Sulfate administration was allowed in this type of indication but only if it did not delay the cesarean delivery.

**Table 2:** Characteristics at birth and neonatal morbidities.

Characteristics at birth and in neonatal period	Magnesium sulfate (n = 94)	No magnesium sulfate (n = 44)	*P* value
Gestational age (weeks)^1^	29.6 +/− 2.1	29.5 +/− 2.2	0.6
Male, n (%)	49 (52.1)	20 (45.5)	0.5
Birthweight (g)^1^	1373 +/− 393.9	1244.7 +/− 403	0.09
Head circumference (cm)^1^	27.7 +/− 2.5	26.7 +/− 2.6	0.06
Mortality, n (%)	3 (3.2)	5 (11.4)	0.06
Severe neurological morbidity^2^, n (%)	6 (6.4)	4 (9.1)	0.6
Mortality + severe neurological morbidity, n (%)	9 (9.6)	9 (20.5)	0.08
Cord pH < 7.10 at birth, n (%)	1 (1.1)	6 (13.6)	<0.01
Apgar scores at 5 minutes < 7, n (%)	11 (11.7)	16 (36.4)	<0.01
Lactates >5 at birth, n (%)	8 (8.5)	10 (22.7)	0.06
Intubation, n (%)	45 (47.8)	18 (40.9)	0.5
External cardiac massage, n (%)	7 (7.4)	8 (18.1)	<0.05
Epinephrine, n (%)	7 (7.4)	8 (18.1)	<0.05
Maternal-fetal infection^3^, n (%)	44 (46.8)	27 (61.4)	0.15
Necrotizing enterocolitis, n (%)	4 (4.3)	1 (2.3)	0.6
Intraventricular hemorrhage (IVH), n (%)	10 (10.6)	6 (13.6)	0.6
Severe IVH (grade 3-4), n (%)	5 (5.3)	4 (9)	0.5
Periventricular leukomalacia, n (%)	1 (1.1)	0	0.5

^1^Values are given as mean +/− standard deviation.

^2^Periventricular leukomalacia and intraventricular hemorrhage grade 3-4.

^3^Defined as the presence of at least two criteria among clinical signs of infection, leukocyte count >30 g/L or neutrophil <5 g/L, presence of bacteria in gastric or blood samples, or C-reactive protein level >20 mg/L.

**Table 3:** Maternal monitoring during magnesium sulfate administration.

	At the beginning of MgSO4 implementation (n = 81)^1^	4h after the MgSO4 implementation (n = 26)^1^	p
Blood pressure, n (%)	51 (63)	19 (73)	0.12
Heart rate, n (%)	59 (72.8)	17 (65.4)	0.42
Respiratory rate, n (%)	21 (25.9)	11 (42.4)	<0.05
Oxygen saturation, n (%)	7 (8.6)	7 (26.9)	<0.05
Patellar reflexes, n (%)	19 (23.5)	15 (57.7)	<0.05

^1^n are different because 55 women delivered within 4 hours.

## References

[b1] Beck S. *et al.* The worldwide incidence of preterm birth: a systematic review of maternal mortality and morbidity. Bulletin of the World Health Organization. 88, 31–38 (2010). 20428351 10.2471/BLT.08.062554 PMC2802437

[b2] Yeargin-Allsopp M. *et al.* Prevalence of cerebral palsy in 8-year-old children in three areas of the United States in 2002: a multisite collaboration. Pediatrics. 121, 547–54 (2008). 18310204 10.1542/peds.2007-1270

[b3] Australian Cerebral Palsy Registrer Group: Report of the Australian Cerebral Palsy Register, Birth Years 1993–2006. Sydney (2013). 10.1111/dmcn.13026 26762930

[b4] Gathwala G. Neuronal protection with magnesium. Indian J Pediatr. 68, 417–9 (2001). 11407156 10.1007/BF02723017

[b5] Burd I., Breen K., Friedman A., Chai J. & Elovitz M. A. Magnesium sulfate reduces inflammation-associated brain injury in fetal mice. Am J Obstet Gynecol. 202, 292–9 (2010). 20207246 10.1016/j.ajog.2010.01.022 PMC2835629

[b6] Lutgendorf M. A. *et al.* Effect of dexamethasone administered with magnesium sulfate on inflammation-mediated degradation of the blood-brain barrier using an *in vitro* model. Reprod Sci. 21, 483–91 (2014). 24077438 10.1177/1933719113503410 PMC3960840

[b7] Mittendorf R. *et al.* Association between the use of antenatal magnesium sulfate in preterm labor and adverse health outcomes in infants. Am J Obstet Gynecol. 186, 1111–8 (2002). 12066082 10.1067/mob.2002.123544

[b8] Crowther C. A., Hiller J. E., Doyle L. W. & Haslam R. R. Effect of magnesium sulfate given for neuroprotection before preterm birth: a randomized controlled trial. JAMA. 290, 2669–76 (2003). 14645308 10.1001/jama.290.20.2669

[b9] Magpie Trial Follow-up Study Collaborative Group. The Magpie Trial: a randomized trial comparing magnesium sulfate with placebo for pre-eclampsia. Outcome for children at 18 months. BJOG. 114, 289–99 (2007). 17166221 10.1111/j.1471-0528.2006.01165.x PMC2063969

[b10] Marret S., Marpeau L., Zupan-Simunek V., Eurin D. & Lévêque-Hellot M. F. For PREMAG trial group. Magnesium sulphate given before very-preterm birth to protect infant brain: the randomized controlled PREMAG trial. BJOG. 114, 310–8 (2007). 17169012 10.1111/j.1471-0528.2006.01162.x

[b11] Rouse D. J. *et al.* A randomized, controlled trial of magnesium sulfate for the prevention of cerebral palsy. N Engl J Med. 359, 895–905 (2008). 18753646 10.1056/NEJMoa0801187 PMC2803083

[b12] Costantine M. M. & Weiner S. J. Effects of antenatal exposure to magnesium sulfate on neuroprotection and mortality in preterm infants: a meta-analysis. Obstet Gynecol. 114, 354–64 (2009). 19622997 10.1097/AOG.0b013e3181ae98c2 PMC2761069

[b13] American College of Obstetricians and Gynecologists. Magnesium sulfate before anticipated preterm birth for neuroprotection. Obstet Gynecol. 115, 669–71 (2010). 20177305 10.1097/AOG.0b013e3181d4ffa5

[b14] Magee L., Sawchuck D., Synnes A. & von Dadelszen P. SOGC clinical practice guidelines. Magnesium sulphate for fetal neuroprotection. J Obstet Gynaecol Can. 33(5), 516–29 (2011). 21639972 10.1016/S1701-2163(16)34886-1

[b15] Vayssière C. *et al.* Prolonged and post-term pregnancies: guidelines for clinical practice from the French College of Gynecologists and Obstetricians (CNGOF). Eur J Obstet Gynecol Reprod Biol. 169, 10–16 (2013). 23434325 10.1016/j.ejogrb.2013.01.026

[b16] French Society of Anaestesiologist and Intensive Care (SFAR). Multidisciplinary management of severe preeclampsia. Experts’ guidelines. Ann Fr Anesth Reanim. 28(3), 275–81 (2009). 19321292 10.1016/j.annfar.2009.02.015

[b17] Ow L. L., Kennedy A., McCarthy E. A. & Walker S. P. Feasibility of implementing magnesium sulphate for neuroprotection in a tertiary obstetric unit. Aust N Z J Obstet Gynecol. 52, 356–360 (2012). 10.1111/j.1479-828X.2012.01434.x 22515404

[b18] Gibbins J. G., Browning K. R., Lopes V. V., Anderson B. L. & Rouse D. J. Evaluation of the clinical use of magnesium sulfate for cerebral palsy prevention. Obstet Gynecol. 121(2 Pt 1), 235–40 (2013). 23344271 10.1097/AOG.0b013e31827c5cf8

[b19] Tan Y. H. & Groom K. M. A prospective audit of the adherence to a new magnesium sulphate guideline for the neuroprotection of infants born less than 30 weeks’ gestation. *Aust N Z J Obstet Gynaecol*. 55, 90–3 (2014). 25307153 10.1111/ajo.12271

[b20] Papile L. A., Munsick-Bruno G. & Schaefer A. Relationship of cerebral intraventricular hemorrhage and early childhood neurologic handicaps. J Pediatr. 103, 273–7 (1983). 6875724 10.1016/s0022-3476(83)80366-7

[b21] De Vries L. S., Eken P. & Dubowitz L. M. The spectrum of leukomalacia using cranial ultrasound. Behav Brain Res. 49, 1–6 (1992). 1388792 10.1016/s0166-4328(05)80189-5

[b22] Bell M. J. *et al.* Neonatal necrotizing enterocolitis. Therapeutic decisions based upon clinical staging. Annals of surgery. 187, 1–7 (1978). 413500 10.1097/00000658-197801000-00001 PMC1396409

[b23] Englesbe M. J. *et al.* Seasonal variation in surgical outcomes as measured by the American College of Surgeons-National Surgical Quality Improvement Program (ACS-NSQIP). Ann Surg. 246(3), 456–62 (2007). 17717449 10.1097/SLA.0b013e31814855f2 PMC1959349

[b24] Royal College of Obstetricians and Gynaecologists. The management of severe pre-eclampsia/eclampsia; National Institute for Health and Clinical Excellence Antenatal Care. NICE clinical guideline 62. London: National Institute for Health and Clinical Excellence (2006).

[b25] Patient safety checklist: magnesium sulfate before anticipated preterm birth. Obstet Gynecol. 120, 432–3 (2012). 22825114 10.1097/AOG.0b013e318268054c

[b26] American College of Obstetricians and Gynecologists. ACOG Practice Bulletin No. 106: Intrapartum fetal heart rate monitoring: nomenclature, interpretation, and general management principles. Obstet Gynecol. 114(1), 192–202 (2009). 19546798 10.1097/AOG.0b013e3181aef106

[b27] Duley L., Gulmezoglu A. M., Henderson-Smart D. J. & Chou D. Magnesium sulphate and other anticonvulsivants for women with pre-eclampsia. Cochrane Database Syst Rev. 11, CD000025 (2010). 21069663 10.1002/14651858.CD000025.pub2 PMC7061250

