# Supplementary material for: Implementation of an antenatal magnesium sulfate protocol for fetal neuroprotection in preterm infants
Source: Sci Rep. 2015 Sep 29;5:14732. doi: 10.1038/srep14732 (PMC4586759; doi:10.1038/srep14732)
Supplement: Supplementary Information [file srep14732-s1.doc]

**Implementation of an antenatal magnesium sulfate protocol for fetal neuroprotection in preterm infants**

Pierre-Emmanuel Bouet, M.D., Stéphanie Brun, M.D., Hugo Madar, M.D., Anne-Laure Baisson, MwF, Véronique Courtay, M.D., Géraldine Gascoin-Lachambre, M.D., Ph.D., Sigismond Lasocki, M.D., Ph.D., Loïc Sentilhes, M.D., Ph.D.

**Appendix. Local protocol for magnesium sulfate administration for fetal neuroprotection in preterm infants**

1. Objective:

To decrease the incidence of cerebral palsy in preterm infants.

1. Indications:

The decision to implement magnesium must be decided by a staff obstetrician in agreement with anesthesiologist or by consensus after the daily morning staff meeting.

- All women with imminent preterm birth between 24 and 33 weeks of gestation.

- Cesarean section before 33 weeks of gestation.

1. Maternal contraindications

- Electrolyte disorders.

- Renal failure.

- Maternal cardiac arrhythmia.

- Myasthenia.

- Administration of calcium channel blockers during the previous 2 hours.

- "Urgent delivery": any maternal or fetal emergency that requires immediate delivery, such as abnormal fetal heart rate tracing, severe antepartum hemorrhage or abruptio placenta.

1. Place of treatment

Treatment must be initiated in the labor room or the operating room.

Reanimation and resuscitation kit must be easily accessible.

Physician must be readily available for potential complications.

The maintenance infusion, after 1 hour of treatment, can be administered in the high-risk pregnancy unit.

1. Treatment details

- 4-g IV loading dose over 30 minutes, i.e. 40-ml infusion of a 0.1 g/ml MgSO4 solution.

- 1 g per hour for maintenance infusion (maximum of 12 hours).

- Stop at birth; if no delivery occurs, stop after 12 hours of infusion.

1. Maternal monitoring

- Respiratory rate.

- Heart rate.

- Blood pressure.

- Oxygen saturation.

- Consciousness.

- Tendon reflexes.

- Diuresis.

Frequency of monitoring:

- before starting the magnesium sulfate treatment.

- 10 min after beginning the loading dose.

- at the end of the loading dose (30 min).

- then, every 4 hours in case of maintenance infusion.

Treatment is to be stopped immediately if any of the following symptoms occur:

- respiratory rate < 10/min.

- hypotension.

- areflexia.

- disorders of consciousness.

- oliguria/anuria.

Symptoms usually regress when magnesium sulfate is stopped and symptomatic treatment started. The antidote is only used in cases of accidental overdose.

1. Potential maternal adverse events
   1. At the beginning of the infusion

- flush, sweats, hot flashes (inform patient).

- nausea, vomiting, headache, palpitations.

- treatment should be stopped in cases of hypotension, low respiratory rate, disorders of consciousness.

- rare cases of pulmonary edema (usually related to a too-rapid infusion of a high dose).

- 1. Signs of overdose

- First signs: decreasing tendon reflexes, respiratory rate and somnolence.

- Then, areflexia, bradypnea, disorders of consciousness.

- Finally, respiratory failure, coma, cardiac arrest.

**Antidote:** intravenous calcium gluconate 10% over 10 minutes, at recommended dosage for hypermagnesemia if signs of overdose are present.

NB: Magnesium sulfate is eliminated by renal clearance. Overdose is extremely rare with the therapeutic doses used except of some cases of renal failure and oligo-anuria.

- 1. Drug interactions

- No effect on coagulation.

- May potentiate effects if associated with calcium blockers.

- Not contraindicated with the different types of anesthesia (general or loco-regional) but hemodynamic effects (vasodilatation) are possible. Loco-regional agents are recommended as a first-line anesthesia. Magnesium sulfate may potentiate non-depolarizing neuromuscular blocking agents in general anesthesia: the dose must be decreased and monitoring is essential +++.

| Magnesium blood level / clinical signs  (mmol/l) | Clinical signs |
| --- | --- |
| 0.8 - 1 | Normal levels |
| 1.7 – 3.5 | Therapeutic levels |
| 2.5 - 5 | EKG modifications (long PQ, QRS widening) |
| 4 - 5 | Diminished reflexes |
| > 5 | Areflexia, bradypnea |
| > 7.5 | Impaired cardiac conduction, respiratory distress |
| > 12 | Cardiac arrest |
